# Correspondence

**Published:** 1869-07

**Authors:** J. G. Ambler, L. D. Rogers, L. S. Straw

**Affiliations:** Committee. 25 West 23d St. New York.; Committee. 25 West 23d St. New York.; Committee. 25 West 23d St. New York.


### CORRESPONDENCE.

---

### ARTICLE V.

Mr. Editor:—

The Committee of Arrangements would respectfully inform the members of and delegates to the Dental Association, that they have effected arrangements with Messrs. Lelands for holding our meeting in Union Hotel Hall, Saratoga Springs, a splendid room admirably adapted for the purpose, which, together with a committee room, they have with their accustomed liberality offered us free of all expense during the day while we are in session. They also purpose to welcome us in a public Hall, which the committee have gratefully assented to.

The committee have also arranged for the accommodation of those who will give, say ten or twelve days notice to the chairman of the committee of their wishes, &c., at Union Hotel at $5 per day, and at Columbia Hotel ( also kept by one of the Lelands) at $3.50 per day. The delegates with their families whether stopping at the Union or Columbia are to have the free use of the parlors and grounds of both Hotels.

The committee have not been able to procure any abatement in the regular charges at these or any other Hotel in consequence of the time being their busiest season. Hoping this arrangement will meet the approval of all, we would express the hope that this will be the longest and most harmonious meeting the Association has ever held.

J. G. Ambler,
L. D. Rogers, } Committee.
L. S. Straw.

25 West 23d St. New York.

